# Impact of Family-Based Economic Empowerment Intervention, Suubi+Adherence (2012–2018) on Multidimensional Poverty for Adolescents Living with HIV (ALWHIV) in Uganda

**DOI:** 10.3390/ijerph192114326

**Published:** 2022-11-02

**Authors:** Darejan Dvalishvili, Fred. M. Ssewamala, Proscovia Nabunya, Ozge Sensoy Bahar, Samuel Kizito, Flavia Namuwonge, Phionah Namatovu

**Affiliations:** 1 Brown School, Washington University in St. Louis, St. Louis, MO 63130, USA; 2 International Center for Child Health and Development (ICHAD), Uganda Office, Masaka 961105, Uganda

**Keywords:** adolescent wellbeing, adolescents living with HIV, HIV care, evidence-based intervention, impact evaluation, family-based multifaceted economic empowerment intervention, multidimensional poverty, poverty reduction, resource-limited settings

## Abstract

Children growing up in poverty are disproportionately affected by diseases, including HIV. In this study, we use data from Suubi+Adherence, a longitudinal randomized control trial (2012–2018) with 702 adolescents living with HIV (ALWHIV), to examine the effectiveness of a family-based multifaceted economic empowerment (EE) intervention in addressing economic instability and multidimensional poverty among ALWHIV in Southern Uganda. We constructed a Multidimensional Poverty Index of individual and household indicators, including health, assets, housing and family dynamics. We computed the proportion of multidimensionally poor children (H), estimated poverty intensity (A) and adjusted headcount ratio (M_0_). Using repeated measures at five-time points (baseline, years 1, 2, 3 and 4-post baseline) across two study arms: treatment (receiving the EE intervention) vs. control arm (not receiving EE), we find that both the incidence and proportion of multidimensional poverty decreased in the treatment arm vs. the control arm. Given that there is a direct link between economic instability and poor health outcomes, these findings are informative. They point to the potential for family EE interventions to decrease multidimensional poverty among vulnerable children, including ALWHIV, impacting their overall wellbeing and ability to meet their treatment needs and improve HIV care continuum outcomes.

## 1. Introduction

An estimated 38.4 million people were living with HIV globally in 2021 [1]; of these, 2.73 million were children under 19 [1]. Nearly 88% (1.71 million) of adolescents living with HIV (ALWHIV) are from sub-Saharan Africa (SSA) [1,2], which is also home to two-thirds of the extremely poor global child population—defined as living on less than US$1.90 PPP (Purchasing Power Parity) a day [3], despite the region only containing 23% of the child population [4]. Due to the specific nature of child/adolescent needs, such as education and care, relying on monetary deprivation as the only criterion to assess child poverty is restrictive [5]. Based on the capability approach, poverty is not just a lack of income to meet basic needs but deprivations of basic human capabilities and freedom to achieve valuable functioning and living conditions [6,7,8].

Evidence from the SSA region indicates that economically vulnerable children are disproportionately affected by HIV [9,10,11,12]. Moreover, despite increased access to antiretroviral therapy (ART), HIV remains one of the leading causes of death among adolescents in SSA [2]. ALWHIV face distinct challenges at all stages of the HIV care continuum, including diagnosis, linking to HIV care services, staying in care and maintaining treatment [13]. Indeed, ART adherence among adolescents remains low and treatment failure rates are comparatively higher than in other age groups [14]. Yet the success of ART depends significantly on a patient’s ability to access treatment and adhere to the required drug regimen [15].

Poverty has been documented to be one of the significant barriers to ART adherence among adolescents [16]. Specifically, ALWHIV living in poverty have limited access to medical resources and struggle with food insecurity and the cost of transportation to clinics for medical refills [17,18,19,20,21]. Overall, people living with HIV, including children living in poor households, encounter greater ART adherence challenges compared to their counterparts living in more economically stable households [22,23].

The association between poverty and HIV has been theorized to be bidirectional [24]. On the one hand, adolescents living in poverty are likely to engage in transactional or age-disparate sexual relationships [25,26] and have limited access to health services [27], increasing their susceptibility to HIV infection. On the other hand, HIV can exacerbate household poverty via higher household expenditure related to medical expenses or loss of income [28]. Thus, it is critical to strengthen the economic and financial stability of poverty-impacted ALWHIV to meet their treatment needs and improve HIV care continuum outcomes.

The emerging literature indicates that multifaceted interventions, such as providing financial resources and incentives to save, together with support services (training, mentorship), have the potential to alleviate deep poverty [29,30,31,32] and address multidimensional poverty among vulnerable adolescents [33]. However, to our knowledge, no research has taken a multidimensional approach to poverty among ALWHIV and examined the impact of a multifaceted economic empowerment (EE) intervention on addressing it among ALWHIV. To fill that gap, this paper examines the effectiveness of a family-based multifaceted economic empowering intervention comprising Child Development Accounts (CDA), microenterprise workshops and mentorship in alleviating multidimensional poverty among ALWHIV.

## 2. Materials and Methods

### 2.1. Data

This paper uses the data obtained from the Suubi+Adherence study. This five-year longitudinal clinical trial (Grant # 1R01HD074949) evaluated the impact and cost-effectiveness of an EE intervention on ART adherence [34]. Participants were drawn from 39 clinics/health centers within the greater Masaka region of Southern Uganda, a relatively poor region heavily affected by HIV and AIDS [35]. A total of 702 adolescents who met the following inclusion criteria were enrolled in the study: (1) ages 10–16 years old; (2) HIV-positive and aware of their status (previously tested for HIV and confirmed by a medical report); (3) prescribed antiretroviral therapy (ART); (4) registered at one of the 39 clinics/health centers for follow- up care and drug refills; and (5) living within families (not institutions).

#### 2.1.1. Randomization and Study Conditions

ALWHIV receiving care at Health care facilities in Southern Uganda participated in the study. Specifically, 39 clinics were randomly assigned to either the treatment arm (*n* = 20 clinics, *n* = 358 participants) or the control arm (19 clinics, *n* = 344 participants). The randomization –done at the clinic level (cluster randomization), was based on the number of children served at the health care facility, location: rural vs. urban and the level of health facility in Uganda’s health system (health care center level IV or III). Hospitals were randomized separately from other healthcare facility categories to balance randomization. The cluster randomization design allowed all participants attending the same clinic to be assigned to the same study condition. This method was intended to minimize contamination or cross-overs.

The medical standard of care (SOC), which all study participants received was comprised of the Uganda Ministry of Health Guidelines for pediatric and adolescent HIV care and treatment, plus information leaflets on adherence and support delivered by lay counselors (including “expert clients”—people living with HIV trained in ART adherence counseling). Due to the inconsistency with which SOC is provided in the study region, the Suubi+Adherence study bolstered the SOC in both the control arm and the treatment arm with eight information sessions on ART adherence using print cartoons to portray adherence topics in a relatable manner [36]. The bolstered SOC was facilitated by a trained research assistant.

In addition to bolstered SOC (described above), received by all study participants (control and treatment), adolescents in the treatment arm received a family-based EE intervention consisting of child development accounts (incentivized savings accounts matched at a rate of 1:1), financial literacy training (FLT), plus microenterprise workshops [34]. The incentivized matched savings and microenterprise were for medical and/or education-related expenses for those ALWHIV in school (including school lunches). The microenterprise workshops included four one-hour FLT group sessions to provide financial management and microenterprise development training to children and their caregivers. In addition to the FLT sessions, treatment arm participants received 12 group-based sessions covering future goal-setting and business development. All components of FLT and microenterprise workshops had been previously proven feasible to implement and acceptable to participants [36].

#### 2.1.2. Data Collection

**Figure A1:**
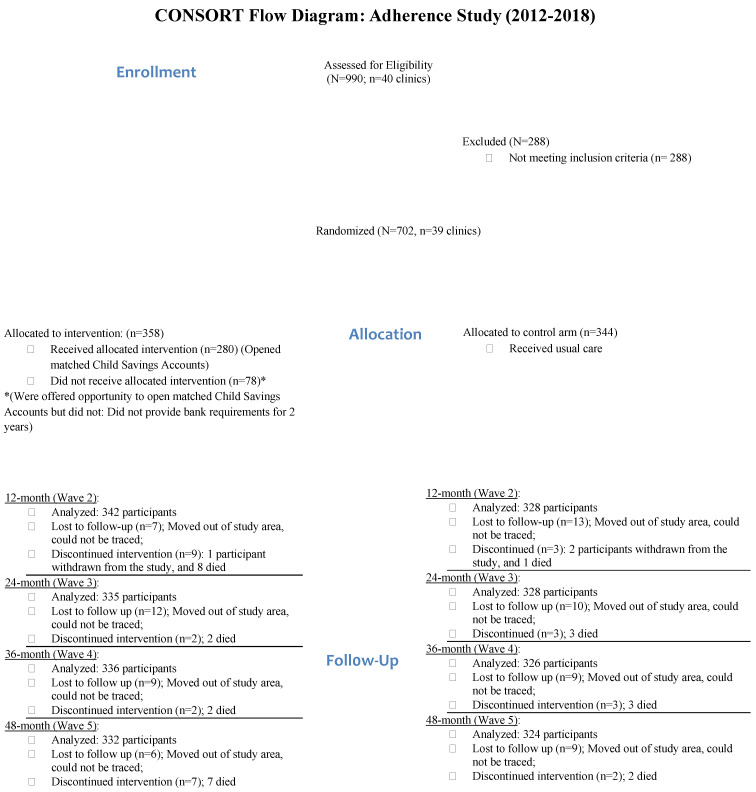
Consort Flow Diagram: Adherence Study (2012–2018).

This paper uses data collected at baseline, 12-, 24-, 36- and 48-months post-intervention initiation. Data were collected between 2012 and 2016 (for more details about enrolment, randomization and attrition, please see the Figure A1: Consort Flow Diagram). The attrition rate at the 48- month post-intervention initiation was 6.55% and was not statistically significantly different across the study arms. Interviewer-administered questionnaires were conducted in Luganda, the commonly spoken language in the study region. The tool combines questions explicitly developed for youths living with HIV and pre-established and validated assessment measures. Participants were assessed on a range of topics: family cohesion, community satisfaction and resources, experience at school, psychosocial concerns, physical health, mental health, medication adherence and drug and sexual risk behavior. The assessment battery was adapted from previous regional studies [37,38,39,40,41,42].

### 2.2. Measures: Multidimensional Poverty Index (MPI)

One way to operationalize the capability approach is by applying a multidimensional poverty index (MPI). The MPI is a poverty measure assessing the extent of deprivation or inability to meet standards of adequate functioning [43]. The methodology allows for the disentangling of the intervention effects, both individually and across the joint deprivations and could be particularly suitable in the developing world [43,44,45,46,47,48]. The method consists of a dual cut-off strategy. After selecting the appropriate indicators, the first cut-off is applied to obtain the dichotomous deprivation in each hand. The second cut-off, the poverty cut-off, is the sum of weighted deprivations an individual has to attain to be considered poor. After the poverty cut-off is applied, the headcount (or incidence) is calculated as the proportion of people considered poor (H). Next, the intensity of poverty (A) and finally, the adjusted headcount ratio of poverty (M_0_) is calculated as the product of H and A. M_0_ is “the sum of the weighted deprivations that the poor (and only the poor) experience, divided by the total population” [43].

To better reflect the specifics of multidimensional child poverty, this paper used the child-centered Multidimensional Overlapping Deprivation Analysis (MODA) approach developed by UNICEF [37]. Using MPI methodology [43], MODA provides a broad approach to the multidimensional aspects of (child) poverty and deprivation, applying the child rights-based approach and using the child as the unit of analysis [46].

The study followed the Global MPI structure that involves selecting a set of indicators, setting the deprivation cutoffs for each indicator, choosing weights for each indicator and then determining a poverty cutoff [43]. We constructed the MPI index considering the Ugandan context by balancing theoretical justifications, empirical evidence and data availability, considering the Child Rights Convention [45,46,47]. The list of selected indicators and dimensions is presented in Table 1. These dimensions cover a set of indicators, including (1) malnutrition, perception of physical health and mental health under the dimension of health; (2) savings, clothing and shoes and means of communications under the dimension of assets; (3) water source, type of housing and access to electricity under the dimension of housing; and (4) child work, family cohesion and school dropout under the dimension of family risk factors.

All indicators, except housing and assets, are defined at the individual child’s level. The indicator for deprivation in physical health uses the cutoff as 1—very poor, poor and/or fair; 0—good or excellent. The indicator for mental health indicates 1—five and a higher score on the CDI inventory and 0—scored below five [48].

Family/household cohesion is measured by a dummy variable using a family cohesion’s Likert Scale (always, most of the time, about half the time, sometimes or never occurs) [49]. In this scale, family cohesion is defined as shared affection, support, helpfulness and caring among family members. Questions and statements included: “Do your family members ask each other for help before asking non-family members for help? Do your family members feel close to each other? We do things together as a family” [49]. Adding household-level characteristics to the MPI was essential to capture the household environment (basic levels of infrastructure and atmosphere), which is critical for healthy child development and wellbeing [46]. However, due to data limitations, some factors, such as exposure to violence and sanitation conditions, were not included in the measurement [43].

Similar to the Global MPI measure and MODA, this study also adopted equal weight across dimensions and equal weight across indicators in each dimension [43,46]. However, as our sample is children living with HIV, we adopted a poverty cutoff mirroring deprivation in two whole dimensions, totaling 2/4 of all indicators. (We tried alternative cutoffs: when the poverty cutoff is 0/4 (a child is not deprived in any domain), the adjusted headcount ratio of children in poverty is 98.6 at baseline and 98.5 at wave 5; when the poverty cutoff is 1/4 (a child is deprived in a total of one dimension), the adjusted headcount ratio of children in poverty is 46.2 at baseline and 38 at wave 5; when the cutoff is 3/4 (a child is deprived in a total of three dimensions), the adjusted headcount ratio of children in poverty is 5.8. at baseline and 4.1 at wave 5. This suggests that 2/4 is a better cutoff that captures variations in multidimensional poverty transitions.) After applying the 2/4 poverty cutoff (children who are poor within two dimensions), we computed the proportion of children who are multidimensionally poor, which is denoted as H (headcount ratio, or poverty incidence) and A—to capture the intensity of multidimensional poverty calculated as the average of deprivation scores among the poor (the sum of weighted deprivation status across indicators.). At the final stage, we calculated M_0_—the adjusted headcount ratio (MPI), calculated as H x A as “the sum of the weighted deprivations that the poor (and only the poor) experience, divided by the total population” [43]. These three indicators (H, A, M_0_) were used to depict multidimensional poverty patterns across groups and time points.

### 2.3. Analysis

The analysis for MPI and its three indicators (H, A, M_0_) was conducted with the -mpi command in STATA 15 [50]. To evaluate the impact of the Suubi+Adherence intervention on multidimensional poverty, the analysis focused on the effects of the intervention on the multidimensional poverty status (1—poor, 0—otherwise). We used Microsoft Excel to create a visual demonstration of the results.

In addition, to explore the impact of the intervention on children, also taking into account their demographics, we used three-level hierarchal models with robust standard error estimation as multiple observations (level 1) across time were nested within each individual (level 2) who were clustered in clinics (level 3) using -melogit command in STATA 15. The fixed effects included the main effect of the intervention, time and the intervention–time interaction. This method enabled us to count for correlation among repeated measures within individuals and any correlations within the same clinics, and to estimate the individual-level random intercepts and the individual random slopes across different time points. We also estimated interclass correlation at both clinic and individual levels. The significance level was set at a *p*-value of 0.05. The model fits were assessed using Likelihood-ratio tests.

## 3. Results

### 3.1. Descriptive Analysis

As shown in Table 2, of the total sample, 56.49% were females with an average age of 12.42 years; 37.23% reported that both parents were alive (non-orphans). In addition, 46.93% reported a parent as their primary caregiver and only 10.73% of the caregivers were employed. The average number of people in the household was six adults and two children under 18. The continuous variables (age, number of HH members, number of children) were compared using a ttest, while categorical variables (sex, orphanhood status, primary caregivers, employment)—using chi-square tests.

### 3.2. MPI Analysis

To calculate the MPI, we measured the poverty level based on the indicators following the AF method [35]. Each of the four domains (health, housing, assets and family factors) was constructed with three binary indicators with the deprivation thresholds defined in Table 3. The bivariate analysis showed no statistically significant observable differences between the control and treatment arms at baseline except for electricity: the analysis showed that 80.81% of the participants from the non-intervention group had electricity, while only 72.63% from the intervention group.

**Figure 1:**
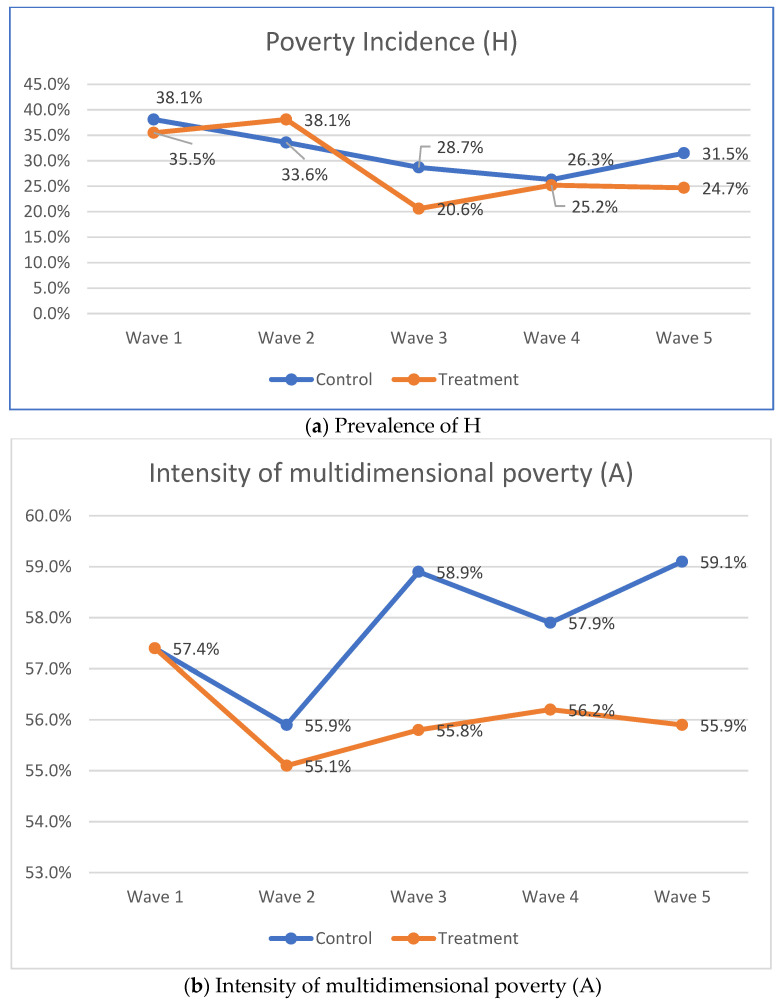

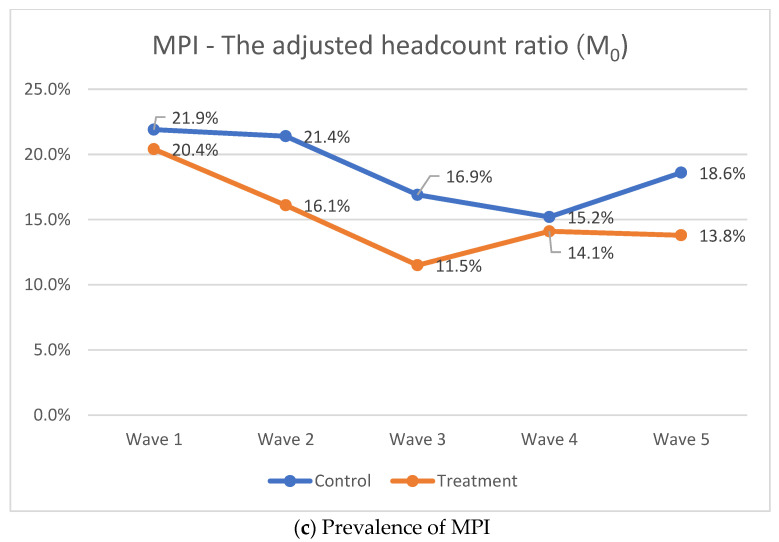
MPI Results by study arms and time points.

To evaluate the impact of the family-based EE intervention, we compared the means of aggregated measures (H, A and M_0_). The results of MPI estimations for H (the headcount ratio), A (the intensity) and M_0_ (MPI—the product of H and A) are presented in Figure 1. The analysis showed that 38.1% of adolescents from the control arm and 35.5% in the treatment arm lived in poverty at baseline (H); poverty (A) intensity was 57.4% for both arms. Overall, 21.1% of all adolescents were multidimensional poor at baseline (M_0_) (Figure 1).

**Figure 2:**
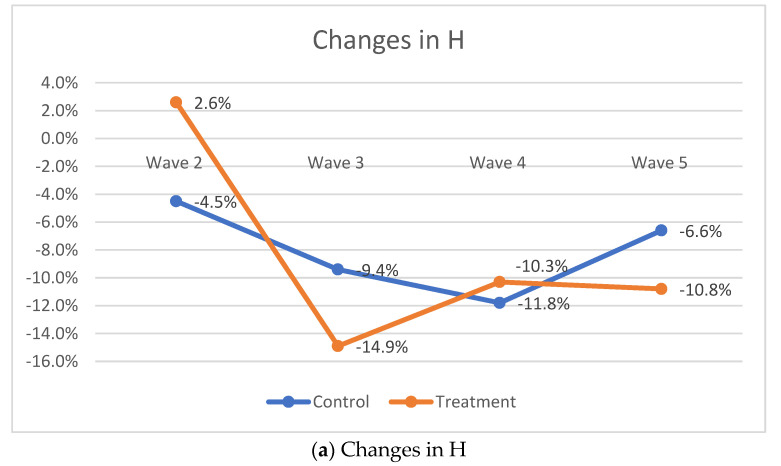

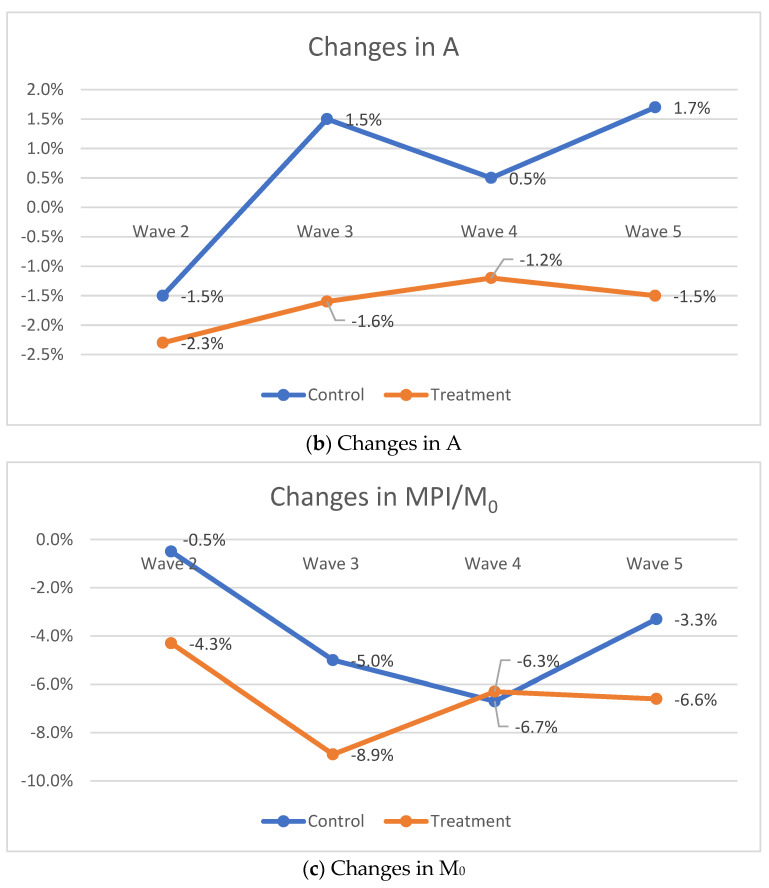
MPI Results: the changes between time points by study arms in percentage points.

At the time the intervention ended (Year 2), the headcount ratio of those in poverty among the treatment arm participants dropped to 20.6% vs. 28.7% for the control arm. Even though the ratio of people in poverty increased by year 4/Wave 5 (which was 2-year post-intervention), the participant from the treatment arm still did better than the adolescents from the control group—24.7% for the treatment arm vs. 31.5% for the control arm. As Figure 2 shows, by wave 4, the poverty incidents increased, but still, by Wave 5, poverty incidence decreased among the treatment group, while it increased among the adolescents in the control condition (Figure 2).

**Figure 3:**
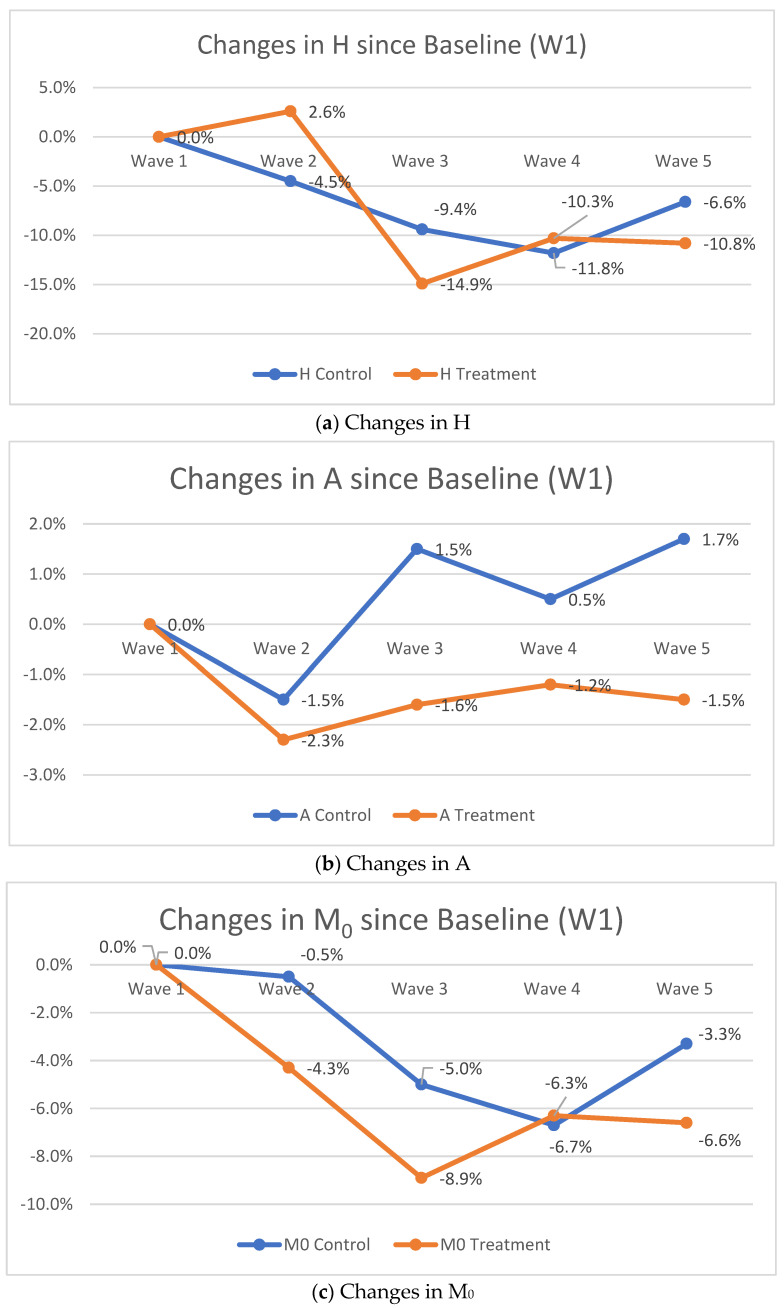
MPI Results: the changes between different time points and baseline in percentage points.

As for poverty intensity, the analysis showed that while poverty intensity increased for adolescents in the control condition from baseline measures, it decreased for the treatment condition by 0.3 percentage points at wave 5 and it increased for the control condition by 1.2 percentage points (Figure 3).

The evaluation of the adjusted headcount ratio (M_0_) indicates that, although multidimensional poverty decreased for both study conditions, it decreased twice as much for the adolescents in the treatment arm vs. participants in the control arm. Specifically, the observable decrease in the treatment arm was by 6.6 percentage points compared to 3.3 percentage points for adolescents in the control condition (Figure 3).

### 3.3. Sensitivity Analysis

To further explore the effect of the Suubi+Adherence intervention, we built multi-level hierarchal models. The binary outcome, MPI poverty incidence, was estimated using a Multilevel mixed-effects logistic regression (MELOGIT) and the results with the odds ratio are reported in Table 4.

Model 1 was built to explore the role of descriptive characteristics (age, gender, family demographics) in multidimensional poverty. The analysis showed that the intervention decreased the odds of being poor by almost 42.8% (*p* = 0.012); specifically, girls faced nearly 27.6% fewer odds of being poor than boys (*p* = 0.048). The caregiver’s relationship with the child/children was not statistically significant. However, caregiver employment decreased the odds of being poor by 65.1% (*p* < 0.000). Furthermore, losing even one parent increased the odds of being poor by 96.4% (*p* < 0.000).

In addition, with each additional year in age, the odds of being poor decreased by 52.8% (*p* < 0.000). However, including the quadratic term in the model showed that this effect does not have a linear relationship. To examine whether the intervention affected children of different ages, we added interaction terms of treatment and age to the model (model 2). In this model, the odds ratio for treatment (OR = 0.681 *p* = 0.605) and interaction term (OR = 0.988, *p* = 0.806) were not significant. Additionally, likelihood-ratio test did not show better fit for the model (LR chi2(1) = 0.06, *p* = 0.806). Thus, that interaction term was dropped in Model 3.

Regarding the intervention’s effect over time (Model 3), dummy variables for time and the interaction of treatment and time were added to the original model. Controlling for time, the intervention was not significant (OR = 0.607, *p* = 0.0803). However, time itself positively reduced the odds of being poor by 54.0% (year 2) and 74.6% (year 4). The model demonstrated a better fit LR chi2(8) = 47.49; *p* = 0.000).

Other variables remained statistically significant, including gender, i.e., girls were 30.2% less likely to live in poverty (OR = 0.698 *p* = 0.027). Caregivers’ employment significantly reduced the risk of living in poverty by 64.3% (OR = 0.357, *p* < 0.0000). Being a single orphan was associated with 91.7% higher odds of living in poverty, while double orphanhood aggravated living in poverty even more (OR = 2.446, *p* < 0.000).

The random effect portion of the models indicated that variance across the levels did not change much across the models. However, it is important to mention that multidimensional poverty is almost not correlated with clinics (ICC = 0.03, CI: 0.01- 0.09), but it is correlated with the same children within clinics—we estimate that children and clinic random effects compose approximately 48% of the total residual variance (ICC = 0.48, CI: 0.42–0.54). We also tested the model with a random intercept and a random coefficient on a child within the clinic level (not shown here). As the Likelihood-ratio test did not indicate significant differences (LR chi2(2) = 5.75, *p* = 0.057) with model 3, we reported the results from a more parsimonious model.

## 4. Discussion

This paper utilized data from a cluster randomized controlled trial (Suubi+Adherence) to examine the impact of a multi-faced, family-based economic empowerment intervention, including a matched savings account, mentorship and financial literacy training, on multidimensional poverty among ALWHIV in southern Uganda. We composed a Multidimensional Poverty Index (MPI) relevant to the needs of ALWHIV and the local Ugandan context. To assess the impact of the intervention, we compared the means of aggregated measures (H, A and M_0_) and used mixed-effect models to explore specifics.

We examined the specifics of the multidimensional poverty among ALWHIV, who, to our knowledge, were not studied in this regard. In addition to the everyday challenges they face related to HIV care [13], poverty imposes additional barriers on adolescents [16]. Thus, it is essential to measure poverty among ALWHIV and find and propose possible strategies to meet their specific needs and decrease their vulnerability.

Our findings suggest that ALWHIV who were orphans were more likely to be poor. Considering the scale of the problem, such as high rates of HIV and that almost 10% of children are orphans in Uganda [51], the findings again emphasize the importance of addressing the needs of this particular group. Our study also showed that multidimensional poverty decreased with time as adolescents got older; however, older adolescents increased the odds of falling into poverty again. These results indicate that these subgroups of ALWHIV, specifically orphans and older adolescents, may need additional help over and above EE to get them out of poverty.

On the other hand, in our sample, girls are doing better than boys. Further research is needed to explore the phenomenon due to their higher vulnerability to poverty [52] and the fact that women in Sub-Saharan Africa are considered a key population [53,54].

In addition to measuring multidimensional poverty in our study population, we evaluated the effectiveness of EE intervention. Our analysis showed that the Suubi+Adherence intervention reduced multi-dimensional poverty incidence in our sample by 10.8 percentage points (30.4%) among participants in the treatment condition versus 6.6 percentage points (17.3%) for the control condition at year four post-intervention initiation. The results also indicated that the percentage of multidimensionally poor children in the treatment arm decreased by twice as much, with 6.6 percent points (32.4%) versus 3.3 percent points (15.1%) for the control condition. In addition, while poverty intensity increased for participants in the control condition by 1.7 percentage points, it decreased by 1.5 percentage points for the treatment condition. Our findings contribute to the growing evidence of the importance of multifaceted EE interventions for ALWHIV to increase their access to and effectiveness of HIV care and support their ART adherence [21,55,56]. As the studies also show, reducing poverty and social protection were associated with lower risks for HIV [57,58,59]. Moreover, since children under 18 have higher incidents of multidimensional poverty and the SSA region has one of the highest rates of multidimensionally poor people [60], the EE interventions might have even greater implications for reducing child multidimensional poverty in the region [33].

Despite numerous strengths, there are a few limitations that should be mentioned. First, we constructed the MPI considering available limited data and the context of the participant children (ALWHIV). The findings would be more comprehensive with additional information on sanitation, immunization, exposure to violence, etc. Secondly, further analysis is needed to inspect the impact of the intervention on the weighted sum of overlapping deprivations.

## 5. Conclusions

Taken as a whole, our study extends the emerging evidence of the impact of multifaced interventions on multi-dimensional aspects of child poverty. Specifically, family EE interventions make noticeable and significant changes to multidimensional poverty among ALWHIV both in short- and long-term perspectives. Thus, our findings contribute to the evidence supporting family-based EE interventions in decreasing poverty even among the most vulnerable groups [29,30,31,32], specifically regarding its multidimensionality [33]. However, future research is needed to explore which specific intervention parts (savings account with savings match, mentorship, financial capability training) produced more potent effects.

Despite the promising findings, more research and specific interventions need to be conducted to alleviate poverty risks for thousands of children living with HIV, including orphans living in Uganda today. Future research should also explore the effect of the intervention on adolescents transitioning into adulthood in more detail. We also call for examination of the impact of the intervention on other vulnerable attributes of ALWHIV, such as abuse, disinheritance and lack of social protection and on any improvements related to physical and mental health outcomes in more detail as well as some of these factors might be driven by poverty [24].

## Figures and Tables

**Table 1:** Multidimensional Poverty Index (MPI) domains and indicators (measures) and Convention on the Rights of the Child.

Dimension CRC Article	Indicator	Deprived If…	Weight
Health (Article 24)	Malnutrition (I)	The child has less than two meals a day OR the child did not eat any meat/fish last week	1/12
	Perception of Physical Health (I) Mental Health (I)	At present time, how would you rate your physical health? The child has moderate to severe depression as measured by the Children’s Depression Inventory (CDI)	1/12 1/12
Assets (Article 17, 27)	Savings (I)	The child does not have money saved anywhere (bank, Savings and Credit Cooperative, with parents/caregivers)	1/12
	Clothing & Shoes (I)	The child has less than two pieces of clothing or no shoes	1/12
	Means of communication (H)	The household does not own any of the following communication means: television, radio and cell phone	1/12
Housing (CRC Article 24, 27)	Water source (H)	The water source is more than 1 km from the household	1/12
	Type of housing (H)	The family house in not a brick house	1/12
	Electricity (H)	The household does not have electricity	1/12
Family Factors (Article 8, 19, 31, 32)	Child work (I)	The child aged below 18 engages in work	1/12
	Family Cohesion (I/H)	Household maintain family cohesion more than half times when child is in need	1/12
	School Dropout (I)	The school-aged child is not currently enrolled in school	1/12

**Table 2:** Sample Demographics at Baseline.

	Non-Intervention Group		Intervention Group		Total		Test	
	N (344)		N (358)		N (702)			
Demographic Characteristics	#/Percent/Mean	SD	#/Percent/Mean	SD	#/Percent/Mean	SD	Test	*p* Value
Age (10–16)	12.38	1.97	12.46	1.98	12.42	1.98	t (699) = −0.5646	0.5725
Female (ref: male)	191 (56.10%)		203 (56.86%)		396 (56.49%)		x^2^(1) = 0.0410	0.8400
Number of members in HH (Range = 2–18)	5.78	2.46	5.69	2.64	5.73	2.55	t(699) = 0.5121	0.6088
Number of children in HH (Range = 0–14)	2.41	1.88	2.28	1.96	2.34	1.92	t (699) = 0.8939	0.3717
Orphanhood status							x^2^ = 0.8009	0.6700
Both parent alive	123 (35.76%)		138 (38.66%)		261 (37.23%)			
Single Orphan	133 (38.66%)		136 (38.10%)		269 (38.37%)			
Double orphan	88 (25.58%)		83 (23.25%)		171 (24.39%)			
Primary caregiver							x^2^ (2) = 4.2878	0.1170
Parents	150 (43.60%)		179 (50.14%)		329 (46.93%)			
Grandparents	102 (29.65%)		104 (29.13%)		206 (29.39%)			
Others	92 (26.74%)		74 (20.73%)		166 (23.68%)			
Caregiver: employed (ref: unemployed)	29 (8.43%)		46 (12.96%)		75 (10.73%)		x^2^(1) = 3.7388	0.0530

**Table 3:** Descriptive Data for deprivation in Multidimensional Poverty Index (MPI) Indicators at Baseline.

	Non-Intervention Group	Intervention Group	Total	Test	
MPI Indicators (Range)	% (N) (Mean/SD)	% (N) (Mean/SD)	% (N) (Mean/SD)	Test	*p* Value
**Health (0–3)**					
Malnutrition	70.06 (241)	66.11 (236)	68.05 (477)	x^2^(1) = 1.3782	0.24
Poor health perception	24.13 (83)	25.70 (92)	24.93 (175)	x^2^(1) = 0.2312	0.63
Moderate or severe hopelessness	32.85(113)	32.12 (115)	32.48 (228)	x^2^(1) = 0.0422	0.837
**Housing (0–3)**					
Distant water sources	20.75 (70)	20.11 (72)	20.23 (142)	x^2^(1) = 0.0061	0.94
Mud house	13.37 (46)	12.01 (43)	12.68 (89)	x^2^(1) = 0.2935	0.59
Electricity	80.81 (278)	72.63 (260)	76.64 (538)	x^2^(1) = 6.5695	0.01
**Assets (0–3)**					
No savings	71.8 (247)	69.83 (250)	70.80 (497)	x^2^(1) = 0.3293	0.57
Few clothing & no shoes	28.49 (98)	27.37 (98)	27.92 (196)	x^2^(1) = 0.1082	0.74
Lack of communication/information means	81.98 (282)	80.73 (289)	81.34 (571)	x^2^(1) = 0.1807	0.67
**Family Factors (0–3)**					
School dropout	12.50 (43)	12.85 (46)	12.68 (89)	x^2^(1) = 0.0193	0.89
Child labor (<18 years old)	13.66 (47)	13.69 (49)	13.68 (96)	x^2^(1) = 0.0001	0.993
Poor Family Cohesion	49.13 (169)	45.81 (164)	47.44 (333)	x^2^(1) = 0.7745	0.379
**Multidimensional deprivation score** **(0–1)**	0.3987 (0.135)	0.3873 (0.137)	0.3929 (0.136)	t(700) = 1.1096	0.27

**Table 4:** Multilevel mixed-effects logistic regression results.

	Model 1					Model 2					Model 3				
Variables	Odds Ratio	Std. Err.	*p*	CI		Odds Ratio	Std. Err.	*p*	CI		Odds Ratio	Std. Err.	*p*	CI	
Treatment group (ref: Control Group)	0.572	0.127	0.012	0.370	0.883	0.681	0.506	0.605	0.159	2.924	0.607	0.175	0.083	0.345	1.068
Treatment group #Age						0.988	0.049	0.806	0.897	1.088					
Treatment#Time (ref: Control group/Baseline)															
Treatment#Time_wave2											0.825	0.228	0.486	0.480	1.418
Treatment#Time_wave3											0.799	0.223	0.422	0.463	1.381
Treatment#Time_wave4											1.265	0.353	0.399	0.732	2.187
Treatment#Time_wave5											0.907	0.257	0.730	0.520	1.580
Time (ref: Baseline)															
Wave 2											0.644	0.137	0.039	0.425	0.977
Wave 3											0.460	0.105	0.001	0.293	0.720
Wave 4											0.341	0.081	0.000	0.214	0.542
Wave 5											0.273	0.070	0.000	0.166	0.451
Age	0.472	0.091	0.000	0.323	0.688	0.474	0.092	0.000	0.324	0.694	0.774	0.166	0.233	0.509	1.179
Age^2	1.022	0.007	0.001	1.009	1.035	1.022	0.007	0.001	1.009	1.035	1.011	0.007	0.101	0.998	1.025
Gender (ref: male)	0.724	0.118	0.048	0.525	0.997	0.723	0.118	0.048	0.525	0.997	0.698	0.114	0.027	0.507	0.960
Number of children	1.045	0.046	0.323	0.958	1.139	1.045	0.046	0.324	0.958	1.139	1.045	0.047	0.329	0.957	1.140
Number of adults	0.949	0.031	0.109	0.889	1.012	0.949	0.031	0.110	0.890	1.012	0.951	0.032	0.133	0.892	1.015
Primary caregiver															
Grandparents	0.889	0.157	0.505	0.629	1.256	0.890	0.157	0.508	0.630	1.257	0.863	0.152	0.404	0.610	1.220
Other	0.867	0.144	0.390	0.627	1.200	0.868	0.144	0.391	0.627	1.200	0.840	0.140	0.294	0.606	1.164
Orphanhood status															
Single Orphan	1.964	0.361	0.000	1.370	2.817	1.967	0.362	0.000	1.371	2.820	1.917	0.351	0.000	1.339	2.744
Double orphan	2.797	0.662	0.000	1.759	4.448	2.801	0.663	0.000	1.762	4.454	2.446	0.577	0.000	1.540	3.885
Caregiver employed	0.349	0.060	0.000	0.249	0.488	0.349	0.060	0.000	0.249	0.488	0.357	0.061	0.000	0.255	0.501
Constant	610.053	874.739	0.000	36.716	10136.390	558.549	825.328	0.000	30.853	10111.620	9.299	14.914	0.164	0.401	215.607
Random Effects															
Variance of Clinic Random Intercepts	0.156	0.096		0.047	0.521	0.155	0.096		0.046	0.521	0.199	0.108		0.069	0.575
Variance of Child’s Random Intercepts	2.922	0.343		2.321	3.679	2.923	0.343		2.322	3.680	2.844	0.331		2.264	3.573
N of observations	3348					3348					3348				
Wald Test	Wald chi2(11) = 97.48					Wald chi2(12) = 97.56					Wald chi2(19) = 139.17				
LR test vs. logistic model:	chi2(2) = 467.72					chi2(2) = 467.43					chi2(2) = 472.63				
Likelihood-ratio test						Chi2(1) = 0.06; *p* = 0.806					chi2(8) = 47.49; *p* = 0.000				
Residual intraclass correlation															
Level	ICC	Std. Err.				ICC	Std. Err.				ICC	Std. Err.			
Clinic	0.024	0.015		0.007	0.078	0.024	0.015		0.007	0.078	0.031	0.017		0.011	0.086
Child within clinic	0.483	0.029		0.427	0.540	0.483	0.029		0.427	0.540	0.481	0.029		0.425	0.537

## Data Availability

Data is available upon the written request sent to Dr. Ssewamala (PI of the study).
